# Inhibitory control and memory in the search process for a modified problem in grey squirrels, *Sciurus carolinensis*

**DOI:** 10.1007/s10071-019-01261-6

**Published:** 2019-04-11

**Authors:** Pizza Ka Yee Chow, Stephen E. G. Lea, Natalie Hempel de Ibarra, Théo Robert

**Affiliations:** 1 Department of Psychology, Centre for Research in Animal Behaviour, University of Exeter, Exeter, EX4 4QG UK; 2 Division of Biosphere Science, Faculty of Env. Earth Science, Hokkaido University, N10W5, Sapporo, Hokkaido 060-0810 Japan; 3 Max-Planck-Institute for Ornithology, Seewiesen, Germany; 4 Graduate School of Agriculture, Tamagawa University, Machida, Tokyo 194-8610 Japan

**Keywords:** Experience, Plasticity, Inhibition, Proactive interference, Innovation, Squirrels

## Abstract

Inhibiting learned behaviours when they become unproductive and searching for an alternative solution to solve a familiar but different problem are two indicators of flexibility in problem solving. A wide range of animals show these tendencies spontaneously, but what kind of search process is at play behind their problem-solving success? Here, we investigated how Eastern grey squirrels, *Sciurus carolinensis,* solved a modified mechanical problem that required them to abandon their preferred and learned solution and search for alternative solutions to retrieve out-of-reach food rewards. Squirrels could solve the problem by engaging in either an exhaustive search (i.e., using trial-and-error to access the reward) or a ‘backup’ solution search (i.e., recalling a previously successful but non-preferred solution). We found that all squirrels successfully solved the modified problem on their first trial and showed solving durations comparable to their last experience of using their preferred solution. Their success and high efficiency could be explained by their high level of inhibitory control as the squirrels did not persistently emit the learned and preferred, but now ineffective, pushing behaviour. Although the squirrels had minimal experience in using the alternative (non-preferred) successful solution, they used it directly or after one or two failed attempts to achieve success. Thus, the squirrels were using the ‘backup’ solution search process. Such a process is likely a form of generalisation which involves retrieving related information of an experienced problem and applying previous successful experience during problem solving. Overall, our results provide information regarding the search process underlying the flexibility observable in problem-solving success.

**Electronic supplementary material:**

The online version of this article (10.1007/s10071-019-01261-6) contains supplementary material, which is available to authorized users.

## Introduction

A problem occurs when obstacles prevent animals from achieving their goal immediately (Duncker 1945 p. 1). When animals encounter a problem, they often seek alternative means to overcome the obstacle (Thorndike 1898 p. 6). Where animals find a successful solution, they will learn it so that it becomes a cognitively economical method for solving the same or a similar problem. However, when the learned or preferred solution becomes unproductive (i.e., ineffective) for the problem, several key behavioural and cognitive adjustments are needed to achieve problem-solving success. These adjustments broadly reflect animals’ cognitive and/or behavioural flexibility, a type of phenotypic plasticity through which individuals are able to adapt their cognitive process and/or behaviour to changes or demands (Cañas et al. 2003; West-Eberhard 2003 pp. 34–55).

Successful solutions learned from previous experience may proactively interfere with solving subsequent similar problems. Accordingly, the first adjustment is to suppress thoughts and learned or preferred behaviours that have become ineffective, because these impede problem solving. One of the mechanisms that is thought to support such adjustment is inhibitory control (Manrique et al. 2013) which can be defined as the ability to withhold a prepotent or learned response. A high level of inhibitory control can be seen when individuals emit few ineffective behaviours (e.g., a previously learned and preferred solution) after a change is introduced. Persistent emission of ineffective behaviours (or perseveration) is expected to hinder subsequent adjustments when individuals are seeking alternative solutions, leading to problem-solving failure. For example, Hrubesch et al. (2009) showed that chimpanzees (*Pan troglodytes*) failed to obtain an out-of-reach food reward because they did not inhibit the preferred but ineffective behaviour, raking or rattling. In another example, Hanus et al. (2011) showed that chimpanzees failed to obtain a peanut inside a familiar clear jar because they were used to pouring water out of the jar rather than pouring water into it, exhibiting a form of functional fixedness. Morgan (1973) showed that rats that had been reared in isolation, a procedure which results in a general loss of inhibitory control, were slower than control individuals to abandon a learned response of pulling an obstacle out of the way when the apparatus was changed so that the response now required was the easier one of pushing.

The second adjustment is to either modify the existing solution or seek alternative solutions to achieve problem-solving success. The ability to seek alternative solutions when the previously learned or preferred solution becomes unproductive has been shown in different animal species including humans. For example, redfronted lemurs (*Eulemur rufifrons*) successfully inhibited and modified part of a learned motor-sequential technique, from pulling and sliding a lid to pulling and raising the lid when opening a feeding box (Huebner and Fichtel 2015). Other examples include human expert chess players who suppress the use of unproductive (familiar but less efficient) solutions and look for productive (unfamiliar but efficient) solutions (Bilalić et al. 2008a), or gorillas (*Gorilla gorilla*), chimpanzees, bonobos (*Pan paniscus*) and orangutans (*Pongo abelii*) which stop inserting a finger into the hole of a piston and instead lift up the movable base of the piston to grasp a grape (Manrique et al. 2013).

Assuming animals get to the point of trying alternative solutions, the observed flexibility in achieving problem-solving success may be driven by different search processes. It may be driven by exhaustive search, whereby individuals search for any knowledge, information, or available resources that may or may not be related to the problem [see review by Wang and Chiew (2008)]. The exhaustive search process is pure trial-and-error in which individuals could show different types of behaviours [known as ‘exploratory diversity’ in Benson-Amram and Holekamp (2012), or ‘motor diversity’ in Griffin et al. (2014)] or use different behavioural sequences to generate a solution regardless of the past effectiveness of those behaviours (Kolodny et al. 2015). The examples given above that involve an exhaustive search process include the orangutans and other great apes manipulating different parts of the piston to obtain the out-of-reach grape (Manrique et al. 2013) and the redfronted lemurs generating different motor techniques to open the food box (Huebner and Fichtel 2015). The exhaustive search process may not guarantee immediate success and is potentially time consuming. However, it allows individuals to obtain information about the problem, and if there is any solution at all, they may eventually find it.

Alternatively, problem-solving success could be driven by using ‘backup’ solution(s) search whereby individuals revisit their past experience of the same task, or of similar tasks (e.g., Birch 1945; von Bayern et al. 2009). The ‘backup’ solution(s) is likely one of the solutions that animals have previously used to achieve some successes in similar contexts. This search process has been mentioned frequently in human studies (e.g., Bilalić et al. 2008a, b), but may also be seen in non-human animals. For example, orangutans returned to using a stick to obtain syrup in a tube when their preferred techniques became unavailable (Lehner et al. 2011). This ‘backup’ solution search process can also be seen in other contexts: for example, human children use finger counting or verbal counting when they cannot directly find the answer to an addition sum (Geary 1994). In these cases, the ‘backup’ search process may well be considered as a form of generalisation, from successful use of the backup solution in the original task, in non-human animal studies. It necessarily involves memory. For example, keas (*Nestor notabilis*) and New Caledonian crows (*Corvus moneduloides*) that have experienced using stick-like tools to successfully retrieve food also used a stick to obtain an out-of-reach food reward in other situations (Auersperg et al. 2011). The use of the ‘backup’ solution may allow animals to avoid unnecessary movement, effort or delays in achieving problem-solving success, provided that the world (or the animal’s corner of it) is not infinitely variable. If individuals are using the ‘backup’ search process when seeking alternative solutions, they would require few attempts to solve a similar problem as the back-up solutions would lead to close to immediate successful problem solving. They would also show limited behavioural variety, or a tendency to employ particular solutions preferentially to solve a modified task. Note that the two types of search process are not mutually exclusive during the course of the problem-solving process; individuals may switch from one process to another depending on whether they obtain a reward.

In two separate laboratory studies, we previously gave two novel food-extraction problems to five Eastern grey squirrels, *Sciurus carolinensis* (Chow et al. 2016, 2017a, also see Fig. S1a and S1b). These problems involved out-of-reach nuts on one of the two lever-ends. The squirrels could manipulate a lever-end using different types of behaviour (e.g., pressing, pushing, tilting). However, to obtain a nut, they were required to cause a nut or a lever to drop by either pushing the lever end if squirrels were close to a nut container (hereafter, the ‘pushing the near-end’ solution) or pulling the lever end if they were far from the nut container (hereafter, the ‘pulling the far-end’ solution). It is important to note that the opposite actions on a lever-end (i.e., ‘pushing the far-end’ and ‘pulling the near-end’ solutions) were ineffective behaviours and thus, led to problem-solving failure. Accordingly, the position of a nut container could provide information for squirrels to which action they should perform during the problem-solving process. The squirrels were tested under three contexts, namely, when they first experienced the problem (i.e., the original task), when they re-experienced the original problem 22 months after the last success (i.e., the recall task), and when they experienced the same problem that was presented in a different apparatus (i.e., the original-generalisation task). At the first trial of the original task in which the squirrels were completely naïve to the problem, squirrels did not show any preference toward pushing, pulling or pulling-pushing behaviours to solve the task (Fig. S2a and S2b). Across the three contexts, the squirrels showed minimal use for the ‘pulling the far-end’ solution to obtain success (Fig. S3, ranged from 2 to 17% use of ‘pulling the far-end’ solution across the three problems with a max. 180 successes per individual). During the very last trial of the original-generalisation task, one of the squirrels used the successful pulling method once, and all other squirrels did not use it. These performances indicate that the squirrels have shown a strong preference to use the ‘pushing the near-end’ solution to achieve successes. Therefore, the presence of levers is likely to induce the pushing behaviours. This information has laid the foundation to examine how squirrels use inhibitory control to achieve problem-solving success in the context where the learned and preferred solution becomes unavailable as well as their search process that leads to problem-solving success.

Here, we gave the same five squirrels a novel mechanical apparatus in which the ‘pushing the near-end’ solution was blocked (hereafter, the modified problem). Like the previous problems, the mechanical problem had levers that the squirrels acted on (see “Methods” for details). The squirrels could emit different types of behaviour on lever ends but only the ‘pulling the far-end’ solution led to problem-solving success. The fact that squirrels had discovered the alternative successful solution but had not applied it extensively in solving the previous problems led to two possible search scenarios:

1. Squirrels may not have stored the non-preferred solution in memory and therefore, they should conduct an exhaustive search when solving this modified problem. If this is the case, then squirrels would emit more behaviours as a result of solving failure. They would also show different types of behaviours after failing to use the (learned and preferred but now) ineffective pushing behaviour to solve the problem. The solution duration to achieve a success would also be similar to or greater than when they first solved the original problem as reported by Chow et al. (2016) or after they acquired the preferred solution as reported by Chow et al. (2017a).
2. Alternatively, limited experience of using alternative successful solutions may still facilitate the recall of task-relevant information when solving the same or a similar problem (e.g., Bird and Emery 2009). Such an alternative successful solution may be considered as generalisation in some problem-solving contexts and it reveals that individuals may have stored the solution and are able to use it as a backup in a similar context. If this is the case, then squirrels would use few behaviours to solve the modified problem. They would also predominantly emit this alternative successful solution when solving the modified problem. The solution duration to achieve a success would be comparable to their last trial of the original-generalisation task in which their preferred solution could lead to problem-solving success, as reported by Chow et al. (2017a).

## Methods

### Ethical note

Squirrels were not food- or water-deprived during the experiment; squirrels’ daily diet included a mixture of fresh vegetable, fruits, mixed dried seeds, and seasonal food such as acorns. Data were collected in July, 2015. We tested each squirrel when they were active in their home cage between 0700-1100 and 1500-1800. All squirrels were treated under the Association for the Study of Animal Behaviour/Animal Behaviour Society guidance. This study was approved by the University of Exeter Ethics Review Group (no. 2012/253).

### Subjects

Five captive grey squirrels (hereafter, Leonard, Sarah, Simon, Arnold and Suzy) that were living in the Animal Cognition Laboratory at the University of Exeter participated in this study. They were adults (mean age 6 years old), either rescued or hand-raised. Simon, Arnold and Suzy were housed individually (cage sized 1.9 × 1.8 × 2.5 m), whereas Leonard and Sarah were housed together (sized 3 × 1.8 × 2.5 m). In each cage, there was a metal sliding door just below the top of the cage. This door connected to one side of the test room through a metal-mesh tunnel. The test room had two cages (each sized 1.5 × 1.8 × 2.5 m) that was separated by a metal-mesh wall. Only one cage was used at one time. In all rooms, temperature was maintained at 19 °C, with 12-h light–dark cycle.

### Apparatus

**Fig. 1:**
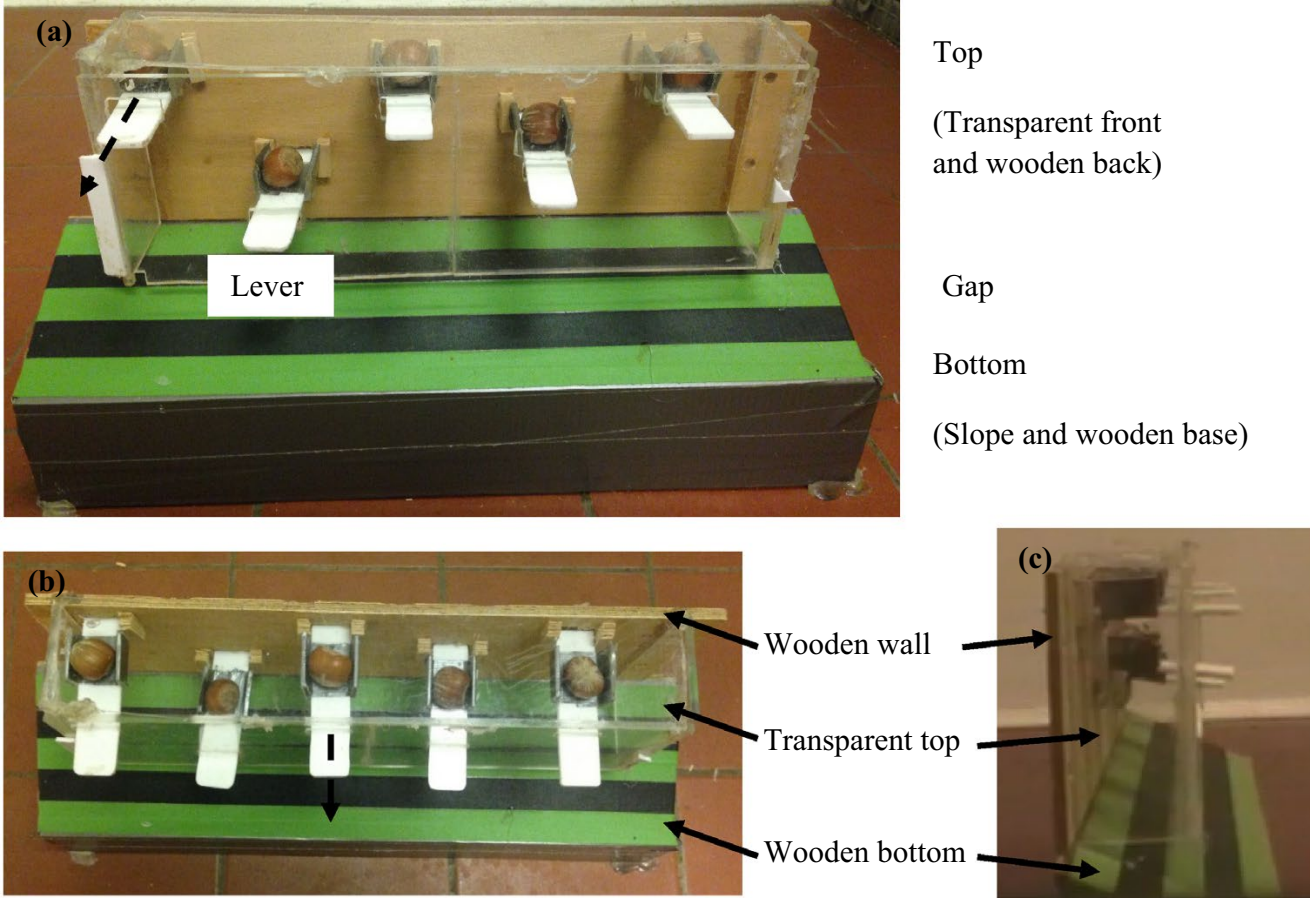
**a** Front view of the modified problem apparatus. **b** Top view of the apparatus. **c** Side view of the apparatus. The apparatus consisted of two parts: the top was made with transparent Plexiglass and the bottom was made with wood. The top part of the apparatus had five holes that were horizontally but not vertically aligned, both on the transparent Plexiglass front and on the wooden back. Each hole had a lever that contained a nut and placed close to the wooden back. The size of a hole was larger than the thickness of the lever so that squirrels were not forced to emit either push or pull behaviours but could also produce other types of behaviours during the solving process. One side of each lever protruded outside the hole so that squirrels could pull it and make the lever and/or the nut drop. The design of the bottom was a sloped platform so that a nut could roll down once the squirrels solved a lever. Dashed arrows indicate the direction of the pull behaviour that squirrels had to emit in order to successfully solve the problem

Figure 1 shows the modified problem for this study. The design of the modified problem was a rectangular-shaped Plexiglass and wooden box. The box could be separated into a top and a bottom part. The top part was a three-sided transparent Plexiglass (30 × 6 × 10 cm, length × height × width) attached to a wooden board (31 × 10 cm), whereas the bottom part of the box (38 × 6 × 10.2 cm) was a wooden slope and stand that supported the box. One side of the top (hereafter, the front) and the wooden board (hereafter, the back) had five rectangular holes that were horizontally but not vertically aligned with each other (each front hole: 2 × 1 cm; back hole: 3.4 × 1 cm). Five levers (each 2.5 × 1.5 cm) with each lever had a three-sided nut container (each 7.8 × 2 × 0.3 cm, length × width × thickness) 1 cm away from one end of the lever were inserted across holes. The lever end with a nut container was placed toward the wooden board horizontally, secured by wooden pillars at the back after being inserted it into the box; this design prevented the pushing solution leading to successful problem solving. The other end of the lever that was far away from the wooden board protruded 1.5 cm outside a hole, which squirrels could act on. To allow squirrels to exhibit different types of behaviours (e.g., tilt up or press) that were not limited to push, pull or consecutive push–pull behaviours [see Table S1 for operational definition in Chow et al. (2016)], the lever was made thinner than the hole (0.3 cm vs 1.0 cm). This design also allowed squirrels to smell and see but not directly reach the nuts. However, only pulling the protruded end of a lever led to successful problem solving. Upon a success, a nut rolled down through the sloped platform (10.2 × 38 cm in green and black colour) which squirrels could obtain the nut through the gap (2.5 cm) between the Plexiglass top and the bottom.

### Procedures

We presented the modified problem to squirrels 6 days after the last food-extraction task that allowed them to use the ‘pushing the near-end’ solution. Squirrels received three blocks of four trials with a 1-day break between blocks (total 12 trials). We presented the modified problem to squirrels once a day. Throughout the experiment, we tested each squirrel individually to avoid any effect of social learning on individuals’ task performance, or the possibility of a dominant individual monopolising the task. Accordingly, each squirrel went into the test room through an over-head metal-mesh tunnel that connected their home-cage to the test room during their active period. In the first trial of the main experiment, we attached the box to the mesh wall so that squirrels could perceive that only one side of the box was ‘functional’. In subsequent trials, we placed the box at the centre of the test room. A trial started when a squirrel went into the test room and ended either when the squirrel completed the task (extracted all five hazelnuts) within 30 min or when they did not respond for 15 min. After every trial, we removed any odour left on the apparatus using cleaning wipes, then the wipes were reapplied after baiting in order to minimise any human scents left on the apparatus. We considered a problem as successfully solved when a squirrel made a lever and/or nut drop. We recorded all behaviours using a video camera (Panasonic SWHD-90) that was attached to the metal mesh of the adjacent test cage.

### Measurements

To understand whether squirrels perceived the modified problem as a novel stimulus, we measured contact latency, the latency to approach an apparatus. We measured the contact latencies for the first trial of the modified problem as well as the first and the last trial of the original-generalisation task (i.e., the very last problem that squirrels were familiar with using the ‘pushing the near-end’ solution). We started to record each latency from the time when a squirrel entered into the test room until the squirrels first touched the apparatus using any of its body parts. If the squirrels perceived the modified problem as a novel stimulus, they would take longer to approach the modified problem than the last trial of the original-generalisation problem. They would also show comparable or higher latency to approach the modified problem than the first trial of the original-generalisation problem as reported in Chow et al. (2017a).

To precisely capture the behaviours that squirrels exhibited during the problem-solving process, we coded all behaviours using a frame-by-frame analysis in PremierePro CS6. Each of the following measurements was taken until a success occurred within a trial (max. 5 successes for each trial). The squirrels completed all trials and they obtained four or more successes on average across trials. However, the fact that they did not obtain all five nuts in some trials (13 out of 60 trials, or 22%, across squirrels) led us to examine performance using trial-by-trial analysis. To do so, we used the average value of each measurement on the trial, by summing each of the values of the measurement on a trial and divided this by the number of levers that a squirrel solved in that trial.

#### Solving attempts

Following the measurements used in Chow et al. (2016, 2017a), an attempt was counted whenever a squirrel touched a lever using any of its body parts, and continued until that body part left the lever. A new attempt was counted if the squirrels touched the same lever or another lever. The total number of attempts to each success incorporated all unsuccessful attempts until a success occurred. It should be noted that the duration of attempts varied and an attempt could include varied number of behaviours or multiple types of behaviours.

#### Solution duration to each success

We summed the durations of attempts (as indicated above) until a success occurred as solution duration. This was recorded as when a squirrel started touching a lever using any of its body parts (squirrels usually used their teeth to bite a lever or nose to touch the lever) until the squirrel left the lever or until a lever/nut dropped.

#### Inhibitory control

The frequency of emitting the learned and preferred but now ineffective pushing behaviour reflected the level of inhibitory control of an individual during the problem-solving process. Accordingly, we recorded the number of ‘pushing’ behaviours, the previously learned and preferred successful solution, to each success. We defined a pushing behaviour as when a squirrel forced a lever moving inward or upward using any of its body parts, but usually its nose, teeth or paws. We considered that the fewer pushing behaviours a squirrel showed, the higher level of inhibitory control it had. Continuous emission of the ineffective pushing behaviour after a failed attempt using such a solution could reflect perseveration (i.e., the persistent used of the ineffective pushing behaviour). We counted the consecutive emissions of the ineffective pushing behaviour before a change to alternative behaviours (regardless of the effectiveness of behaviours) and noted the frequency with which one, two, three, or more than three ineffective pushing behaviours were made before a change to a different kind of behaviour. We considered that a change after emitting one ineffective pushing behaviour reflected a low level of perseveration.

#### Search process

The type of search process used by a squirrel could be deduced from the number of behaviours and the number of types of behaviour that the squirrel employed during the problem-solving process. Specifically, if a search process involved a wide range of behaviours, a squirrel was more likely using the exhaustive search than the backup solution search. In this modified problem, squirrels could emit eight types of behaviours other than pulling. These behaviours included push in, push up, press, tilt up, lick, claw, shake and any combination of these behaviours (see supplementary materials for operational definition of behaviours). We counted the number of types of behaviours and the frequency of each type that squirrels employed until a success occurred.

### Data analysis

We used a one-tailed Wilcoxon signed-rank test to compare the latency to approach the apparatus in the first trial of the modified problem with the first and the last trial of the original-generalisation problem. We used generalised estimating equations (GEE) with exchangeable working correlation matrix to compare the solving duration of the first trial in the modified problem with the solving duration of the first trial in the original problem (Chow et al. 2016), and the solving duration of the last trial in the generalisation problem (Chow et al. 2017a) in which squirrels showed a preference to use the pushing the near-end solution. GEE with Gaussian distribution was used to model the variations of seven behavioural measures: the mean solving duration to each success across trials, the mean number of solving attempts to each success, the mean number of ineffective pushing behaviours to each success, the mean number of consecutive emissions of the ineffective pushing behaviour (i.e., perseveration) before reaching a success, the mean number behaviours to each success, the mean number of behavioural types (including pushing) that squirrels used to solve the modified problem, and the mean number of other behavioural types (excluding pushing) that squirrels emitted after a failed attempt to achieve a success. Because of our small sample size, we adjusted the error variance (Wang and Long 2011) using the package ‘geesmv’ (Wang 2015). All results reported here are two-tailed and we considered a test as significant when *P *< 0.05. All data were analysed using R 3.5.2 (R Core Team 2018).

## Results

### Contact latency in the first trial and solving duration in the modified problem

All squirrels took longer to approach the apparatus in the first trial of the modified problem (median latency = 20 s) than in the last trial of the original-generalisation problem (median latency = 6 s) and this difference was significant (Wilcoxon signed-rank test: *W* = 15, *P* = 0.031). Their first latency to approach the apparatus in the modified problem was comparable to the first trial of the original-generalisation problem (median latency = 23 s, *W* = 6, *P* = 0.406). These results indicated that the squirrels perceived the modified apparatus as a novel stimulus.

**Fig. 2:**
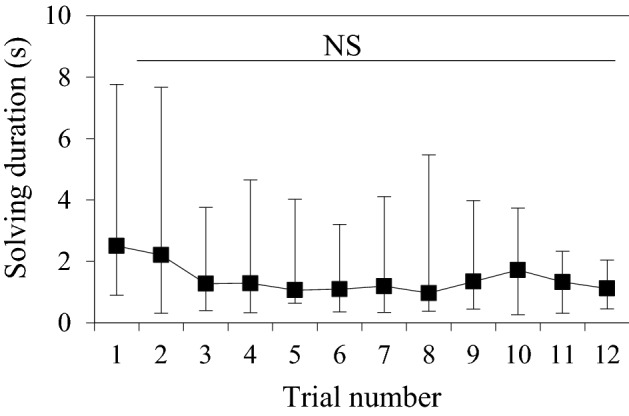
Median, maximum and minimum of mean solving duration to each success in seconds across squirrels over the 12 trials for the modified problem. *NS* not significant

All squirrels solved the modified problem on their first trial (see supplementary video). The median of mean solving duration to each success across squirrels was 2.5 s in the first trial of this modified problem, as compared with 8 s for the first trial of the original problem reported in Chow et al. (2016) and 1 s for the last trial of the original-generalisation problem reported in Chow et al. (2017a). The mean solving duration to each success in the modified problem was significantly lower than the first trial of the original problem (GEE: *χ*_2_^2^ = 7.59, *P *= 0.006), but not significantly different from the last trial of the generalisation problem (*χ*_2_^2^ = 0.68, *P *= 0.411). Figure 2 shows the median of mean solving duration to each success, including the first trial and subsequent 11 trials, for the modified problem. Solving duration did not vary significantly across trials in the modified problem (GEE: *χ*_1_^2^ = 1.63, *P *= 0.202).

### Solving attempts and inhibitory control across trials in the modified problem

**Fig. 3:**
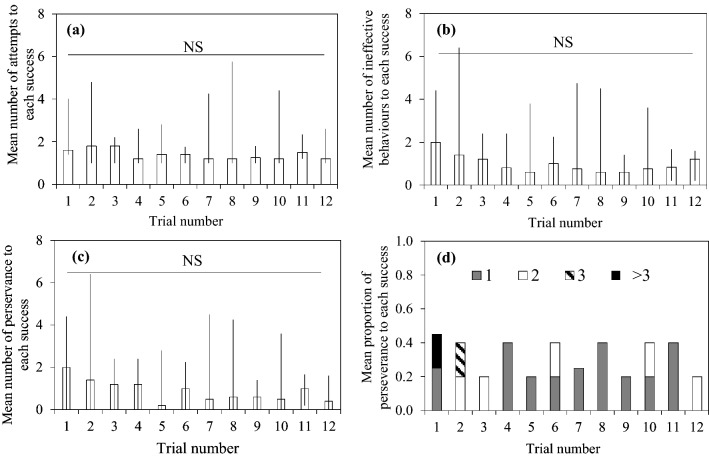
Median, maximum and minimum of **a** mean number of attempts to each success across squirrels over the 12 trials, **b** inhibitory control, measured as the mean number of ineffective pushing behaviours to each success, over the 12 trials, **c** mean number of perseveration, measured as the frequency of consecutive emission the ineffective pushing behaviour before each success, **d** mean frequency in proportion of perseveration categories of ‘1’, ‘2’, ‘3’, ‘> 3’ ineffective behaviours before each success. *NS* not significant

Figure 3a shows the variation of the median of mean solving attempts to each success across trials. In this modified problem, problem-solving success could be achieved with one attempt (i.e., by pulling a lever end). We found that the median of mean number of attempts to each success across squirrels was 1.6 on the first trial. This low number of attempts did not vary significantly across trials (solving attempt: *χ*_1_^2^ = 0.26, *P *= 0.608).

Figure 3b shows the variation of inhibitory control, the frequency of emitting the ineffective pushing behaviour to each success, across trials in the modified problem. On the first trial, the median of mean number of ineffective pushing behaviours to each success across squirrels was 2. This low frequency of emitting the ineffective pushing did not vary significantly across trials (*χ*_1_^2^ = 0.67, *P *= 0.415). Figure 3c shows the variation of perseveration (consecutive emissions of the ineffective pushing behaviour) before reaching a success. On the first trial, the median of mean number of perseveration was 2, indicating that squirrels emitted twice the ineffective pushing behaviours before changing to other types of behaviours. We found perseveration did not vary significantly across trials (*χ*_1_^2^ = 0.42, *P *= 0.517) and squirrels predominantly emitted the ineffective pushing behaviour once or twice in a row (Fig. 3d).

### Search process in the modified problem

**Fig. 4:**
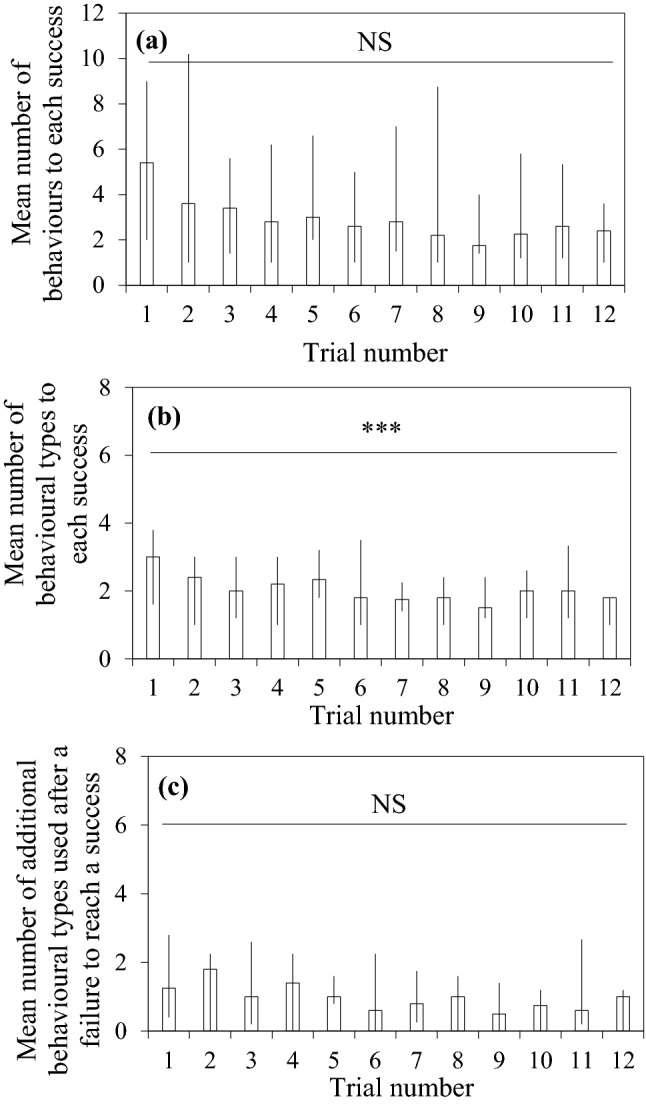
Median, maximum and minimum of **a** mean number of behaviours to each success across trials **b** mean number of different behavioural types across trials that squirrels emitted for the modified problem and **c** mean number of different behavioural types that squirrels emitted after a failed attempt using the pushing behaviour across trials. ***P < 0.001, *NS* not significant

Figure 4a shows the median of mean number of behaviours to each success across trials. The squirrels used 5 behaviours to achieve each success on their first trial and this figure did not vary significantly across trials (*χ*_1_^2^ = 1.73, *P *= 0.189). Figure 4b shows the median of mean number of behavioural types to each success across the 12 trials. Of the nine types of behaviour (including the ineffective pushing behaviour and pulling, the only effective solution) that could be emitted during the problem-solving process, the squirrels used three types of behaviour to reach a success on the first trial (Fig. S4 shows individual data across trials). Across the 12 trials, there was a significant variation in the mean number of behavioural types that squirrels used to solve the modified problem (*χ*_1_^2^ = 64.97, *P *< 0.001); there was a decrease in the mean number of behavioural types across trials. When we excluded the pushing behavioural types to understand how many types of behaviours squirrels used to solve the modified problem, we found squirrels mostly exhibited one type of behaviour to reach a success across trials (*χ*_1_^2^ = 0.34, *P *= 0.562, Fig. 4c); this suggests that squirrels changed from pushing to pulling or combined behaviours that contained pulling, the only effective solution, upon a failed attempt.

## Discussion

When a learned and preferred solution becomes ineffective, two critical adjustments that indicate cognitive/behavioural flexibility are inhibiting ineffective thoughts and behaviours and searching for alternative solutions (Manrique et al. 2013). In the present study, we gave grey squirrels a modified problem that required them to abandon their learned and preferred solution. We found that all squirrels solved the task on their first trial. They required few attempts (Fig. 3a) and emitted few ineffective behaviours (Fig. 3b). They also quickly sought an alternative solution upon failure (Fig. 3c, d). Such adjustments demonstrate their cognitive and behavioural flexibility, which is likely the key contributor to the high solving efficiency on the first and subsequent trials for this modified problem (Fig. 2). These results add to the evidence for how animals other than humans (Bilalić et al. 2008a), great apes (Lehner et al. 2011; Manrique et al. 2013), lemurs (Huebner and Fichtel 2015) and crows (Auersperg et al. 2011) adjust their behaviour under a changed condition.

Memory of using preferred or learned solutions in the past to achieve problem-solving success may proactively interfere with individuals’ flexibility in seeking alternative solutions. A crucial cognitive mechanism supporting the inhibition of ineffective thoughts or behaviours in the current or similar paradigms is inhibitory control. Low levels of inhibitory control are frequently associated with problem-solving failure (e.g., Hanus et al. 2011; Hrubesch et al. 2009). High levels of such control appear to be particularly important immediately after a change occurred. In our case, the learned and preferred solution would induce proactive interference with squirrels’ flexibility to seek alternative solutions, but they mostly emitted only one or two ineffective pushing behaviours on average to each success followed by a quick change to employ alternative behaviours (Fig. 3c, d). These results suggest that proactive interference had minimal effect on the squirrels’ performance; the squirrels quickly realised that the previous solution had become ineffective and showed a high level of inhibitory control by not persistently emitting it when solving the modified problem. With this in mind, the influence of past memory on task performance and the need to use inhibitory control to facilitate learning are also essential for tasks such as discrimination-reversal learning task (Shettleworth 2010 p. 210–211); individuals are required to suppress the learned response toward a rewarded stimulus when it becomes unrewarded in the reversal learning phase. While it is not entirely clear whether a similar form of inhibitory control is involved in solving the modified mechanical problem and discrimination-reversal learning task, it is likely the case because both paradigms involve unlearning a previous reward association.

Our core interest was in the search process behind solving the modified problem. In the introduction, we described two types of search: an exhaustive search during which individuals could employ all relevant and irrelevant knowledge, or emit combinations of different behavioural sequences to solve the task (Kolodny et al. 2015), and a backup solution search during which individuals could revisit task relevant information and alternative solutions that were successful to solve the same or similar tasks. One difference between the two types of search can be seen in the types of behaviours that squirrels employed to solve the task, with exhaustive search involving various types of behaviours, whereas the back-up solution search involves emitting a limited range of behaviour. When the squirrels first experienced the original problem, they exhibited a range of behaviours (six out of nine types of behaviours), which indicated that their search process was exhaustive and learning was trial-and-error (Chow et al. 2016). However, the squirrels did not use diverse behavioural types to solve the modified problem (Fig. 4b, c); they mostly used the previously alternative successful solution (i.e., ‘pulling the far-end’ solution) even though they had little experience of using it when solving the original and the original-generalisation problem. Another difference between the two types of search would be seen in the number of behaviours used to solve a problem. That is, with exhaustive search it is expected to induce a high number of behaviours to each success as individuals are not familiar with the solution, whereas the backup solution search would only require the individuals to use a limited number but accurate behaviours. In our case, the squirrels showed a high number of behaviours in the face of the original problem (Fig. S5), whereas they only emitted a few behaviours (Fig. 4a) or 1–2 attempts to achieve a success in this modified problem (Fig. 3a). These results suggest that squirrels were familiar with the alternative solution and thus, were using the backup solution search.

Logically, the backup solution search would be a better option than the exhaustive search when individuals are solving problems like the modified problem because it increases efficiency to obtain a reward. However, regardless of solving efficiency, both kinds of search processes are viable during problem-solving and each reflects cognitive and behavioural flexibility in a different way. For example, behavioural diversity in the exhaustive search process reflects motor flexibility (Griffin et al. 2014), whereas accurate despite limited behavioural types in the backup solution search process reflect memory flexibility. Nevertheless, the kind of search process may reveal the cognitive processes, if any, that are involved in the observable flexibility to achieve problem-solving success. The use of the backup solution search likely demonstrates generalisation to the modified problem. Given that squirrels showed a high tendency for pulling behaviours, they may have generalised their past successful experience in using the ‘pulling the far-end’ solution to solve the task. Interestingly, squirrels had not used this alternative successful solution extensively before (Fig. S3), but the ability to recall relevant task information without receiving extensive experience has been shown in other species such as rooks, *Corvus frugilegus* (Bird and Emery 2009). One possible explanation for such an ability is squirrels’ ecological foraging characteristics, in that squirrels are generalists (Koprowski 1994) and energy maximisers (Smith and Follmer 1972), so any relevant information that is related to their highly preferred food is expected to lead to a swift learning. This, coupled with our previous finding showing that they are able to remember task relevant information for an extended period (Chow et al. 2017a) may explain their high performance in this modified problem in spite of the limited use of pulling as alternative successful solution in the past.

The fact that squirrels spontaneously solved the modified problem with high efficiency might be explained more simply: they might have a preference to switch to certain type(s) of behaviour in solving this kind of mechanical problem. For example, in the trap-tube task in which animals have to retrieve an out-of-reach reward by avoiding the food falling into a trap, great apes (Martin-Ordas et al. 2008; Mulcahy and Call 2006) and woodpecker finches, *Cactospiza pallida*, prefer pulling to pushing a reward (Tebbich and Bshary 2004). In our case, squirrels might have a preference to switch to pulling. However, our results indicate that no such switch preference was obvious at the beginning when the squirrels first experienced the original task (Fig. S2). More importantly, the fact that pulling a lever end that was close to the nut container led to failure in all past food-extraction problems makes this simpler explanation unlikely. Alternatively, the high preference for using the alternative successful solution for the modified problem may be attributed to squirrels’ attention to task characteristics. Specifically, pulling a lever end that was far from the nut container was the only successful solution when using this side of the lever in the previous problems. Such a solution required the squirrels to pay attention to which end of the lever they were pulling. In the modified problem, the rewards were positioned far from the lever end, which may have facilitated the recall of the relevant solution as well as the squirrels’ solving efficiency. Attention to relevant task information that facilitates problem-solving success has been shown in a wide range of cognitive tasks. For example, attention to which trap is open or closed in the trap-tube task (Martin-Ordas et al. 2008), to which hook is functional in tool use (e.g., Birch 1945; St. Clair and Rutz 2013) and to which string is connected to food reward in string pulling (e.g., Hofmann et al. 2016; Werdenich and Huber 2006) improved problem-solving efficiency. As the squirrels had some previous successful experiences using the pulling the far-end solution (Fig. S3), and strong inhibitory control for using the “pushing the near-end” behaviour, it is possible that they had learned which side of the lever allowed them to emit effective pulling behaviours in their search. The efficiency of recalling a relevant solution may be related to the amount of successful experience that an individual received (Fig. S6) but further investigation is needed to support this hypothesis.

To conclude, we provide evidence for how squirrels solve a problem after their learned and preferred solution becomes obsolete. Although our sample size is small and generalising the results to the whole species may require caution, all the squirrels successfully solved the modified problem on the first trial. They showed two adjustments that underlie flexibility in problem solving: inhibitory control of the ineffective pushing behaviour and switching to alternative behaviours upon failures. When squirrels switched to alternative behaviours, their search process revealed the use of a ‘backup’ solution. The successful recall of an alternative successful solution in spite of limited experience of using it may be facilitated by the characteristics of the task. These results, along with the findings that grey squirrels suffer from minimal proactive interference in this problem-solving task and quickly adapt their behaviour when a change occurs (Chow et al. 2015, 2017b) as well as possess long-term retention of a highly experienced successful tactics in problem solving and transfer these tactics to a similar situation (Chow et al. 2017a), suggest that squirrels are highly flexible in problem solving (Chow et al. 2016). Such flexibility is one key component of complex cognition (Emery and Clayton 2004). But to what extent they are as flexible as other species in this and other cognitive areas remains largely unclear. Further investigations in the area of comparative cognition will not only highlight the similarities and differences between species but will also advance the understanding of the evolution of cognition.

## Electronic supplementary material

Below is the link to the electronic supplementary material.

Supplementary material 1 (AVI 6831 kb)

Supplementary material 2 (DOCX 2543 kb)
