# Supplementary material for: Inhibitory control and memory in the search process for a modified problem in grey squirrels, Sciurus carolinensis
Source: Anim Cogn. 2019 Apr 11;22(5):645–55. doi: 10.1007/s10071-019-01261-6 (PMC6687685; doi:10.1007/s10071-019-01261-6)
Supplement: Supplementary file 2 — Supplementary material 2 (DOCX 2543 kb) [file 10071_2019_1261_MOESM2_ESM.docx]

Supplementary material for manuscript titled as ‘Inhibitory control and memory in the search process for a modified problem in grey squirrels, *Sciurus carolinensis*’. By Chow PKY, Lea SEG, Hempel de Ibarra N, Robert T

Table S1 Operational definitions for coding the behaviours in the original task. We adopted these operational definitions for this study. Direct push in and push up a lever are considered as ineffective behaviours for the modified problem.

| Behaviours |  | definitions |
| --- | --- | --- |
| Identifying a nut | | A squirrel orients its head towards a lever and sniffs within 0.5 cm of a lever more than 1 seconds. |
| An attempt |  | A squirrel uses any of its body part including nose, mouth, teeth, tongue, paw or chin to contact a lever. |
| Pull | Correct* | A squirrel uses its teeth to make an *outward* movement and a lever subsequently moves outside the box.  This pulling behaviour must be performed on the *near* end of the nut container. |
|  | Incorrect# | A squirrel uses its teeth to make an *outward* movement and a lever does not move out of the box.  This pulling behaviour must be performed on the *far* end of the nut container. |
| Push | Correct* | A squirrel uses any of its body part, including nose (usually), teeth, paw or chin to make an *inward* movement of a lever and the lever subsequently moves inside the box.  This pushing behaviour must be performed on the *near* end of the nut container. |
|  | Incorrect# | A squirrel uses any of its body part, including nose (usually), mouth, teeth, paw or chin to make an *inward* movement of a lever and the lever would not moves.  This pushing behaviour must be performed on the *far* end of the nut container. |
| Push up |  | A squirrel uses its nose to make a push under an end of a lever. |
| Push down |  | A squirrel puts force on a lever end with its paws or teeth. This behaviour makes the lever appears in a curve shape. |
| Tilted up |  | A squirrel uses its nose to level up a lever end. This behaviour makes a lever turns 45 degrees. |
| Claw |  | A squirrel uses it front paws to scratch a lever end. |
| Lick |  | A squirrel uses its tongue to touch a lever end. |
| Shake |  | A squirrel uses its teeth to bite a lever end and makes an up-and-down movement. |
| Combined behaviours | | At least two of the behavioural types that mentioned above appear. |

* indicated as direct effective behaviours.

# indicated as non-effective behaviours.

Reference: Chow PKY, Lea SEG, Leaver LA (2016) How practice makes perfect: the role of learning, flexibility, and persistence in problem solving efficiency. Anim Behav 112:273-283. [doi: 10.1016/j.anbehav.2015.11.014](https://doi.org/10.1016/j.anbehav.2015.11.014)

**Fig. S1** **a** The original problem as reported in Chow et al. 2016. This puzzle box was also used in the recall task as reported in Chow et al. 2017 **b** The original-generalisation problem as reported in Chow et al. 2017.


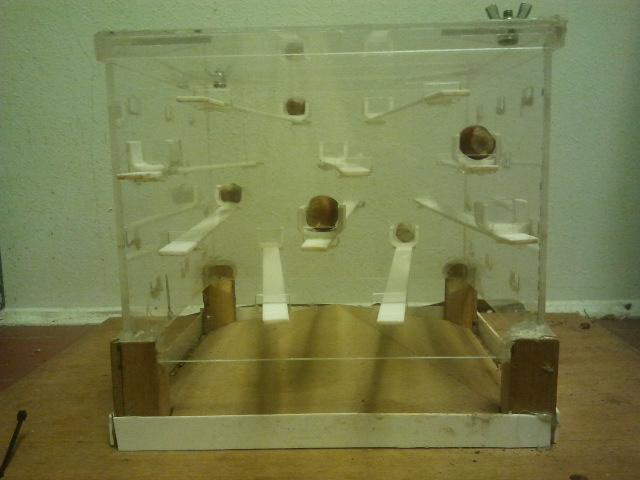


Near end

Base

Legs

Far end

Non-functional levers

Functional levers


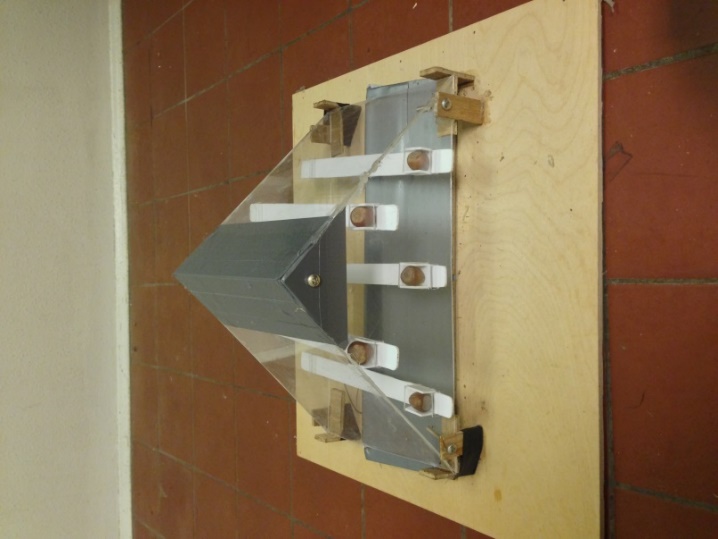

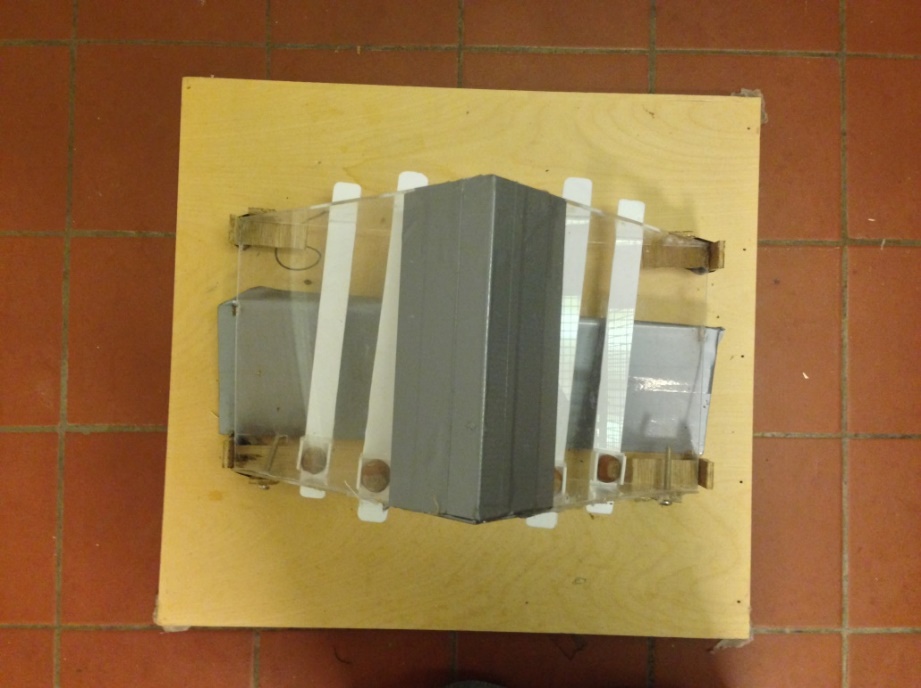


**(b)**

**(a)**

Reference:

Chow PKY, Lea SEG, Leaver LA (2016) How practice makes perfect: the role of learning, flexibility, and persistence in problem solving efficiency. Anim Behav 112:273-283. [doi: 10.1016/j.anbehav.2015.11.014](https://doi.org/10.1016/j.anbehav.2015.11.014)

Chow PKY, Lea SEG, Hempel de Ibarra N, Robert T (2017) How to stay perfect: the role of memory and behavioural traits in an experienced problem and a similar problem. Anim Cog 20:941-952. doi: 10.1007/s10071-017-1113-7

*Analysis on switch preference*

To understand whether squirrels showed a preference for using a particular behaviours to solve this kind of mechanical problems, we analysed ‘switch preference’ which was measured as the frequency of pulling behaviour on the first trial of the original problem in Chow and colleagues (2016); in that study, the squirrels were completely naive to the problem and thus, presuming the effect of learning and experience was minimal. Figure S2a shows three categories of switches of behaviour upon failure, including ‘to pull’, ‘to push’, and ‘to others’. Figure S2b shows the proportion of frequency for each switch category. No squirrels showed a significant difference among the three switch preferences (pooled *χ*^2^_10_ = 10.38, *P* > 0.05).

For each individual, we used Chi-square goodness-of-fit test to examine whether the frequencies of switch preference were equally distributed among the three categories (see below for details). We then pooled the *P* values using Fisher’s formula χ^2^ = −2 Σ In(P) (Sokal and Rohlf 1995 p. 794) across individuals to obtain a chi-square value and compared this value with the corresponding critical value of 10 degrees of freedom.


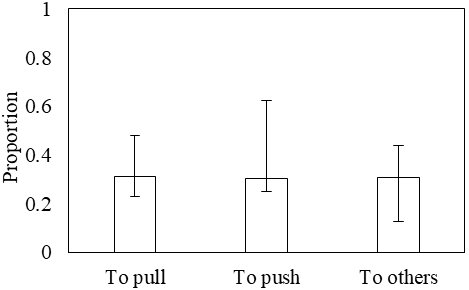


NS

**(b)**

A

A

B

B

C

C

**(a)**

**Fig. S2 a** Schematic illustrating squirrels’ behavioural switch preference for pulling behaviours. Paths A (‘to pull’) include all frequencies of push-pull and others-pull behaviours (dotted arrows). Paths B (‘to push’) include all pull-push and others-push behaviours (thin arrows) and Paths C (‘to others’) include push-others, pull-others and others-others behaviours (solid arrows). **b** On the first trial of the original problem reported by Chow et al. (2016), median, maximum and minimum proportion of change under the three categories: ‘to pull’, ‘to push’ and ‘to others’. NS indicates not significant.

Reference

Sokal RR, Rohlf FJ (1995) Biometry: the principles and practice of statistics in biological research, 3rd ed. W.H. Freeman, New York

*Analysis on previous successful pulling experience*

We further considered how the use of the alternative successful pulling solution in previous experience may have affected squirrels’ search process. To do so, we counted the number of successes for which the squirrels used the ‘pulling the far-end’ solution in the three food-extraction problems that they had previously experienced, namely the original task (Chow et al. 2016), the recall task and the generalisation tasks (Chow et al. 2017a). Overall, the proportion of successes involving the pulling solution was very low (mean proportion = 8.4%).

**Fig. S3** Proportions of successes for which the squirrels used the ‘pulling the far-end’ solution during all previously experienced problems (the number above each bar indicates the actual number of success), namely the original problem (Chow et al. 2016), and the recall task and the generalisation task (Chow et al. 2017a).


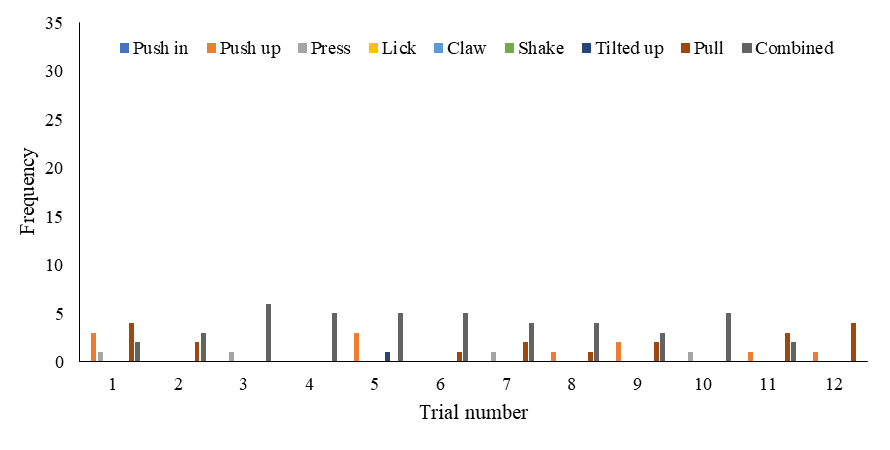
**Fig. S4.** Individual data on the frequency of each behavioural type across trials. Note that these data include all frequencies across successes in each trial.

Leonard


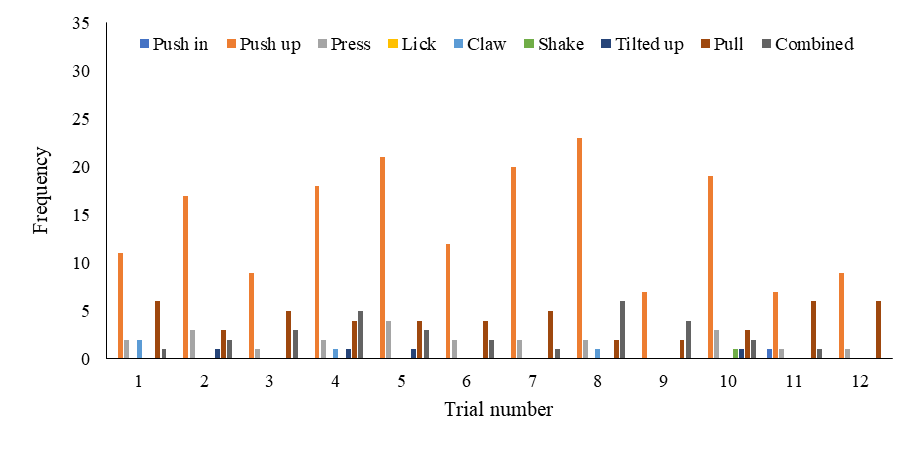
Suzy


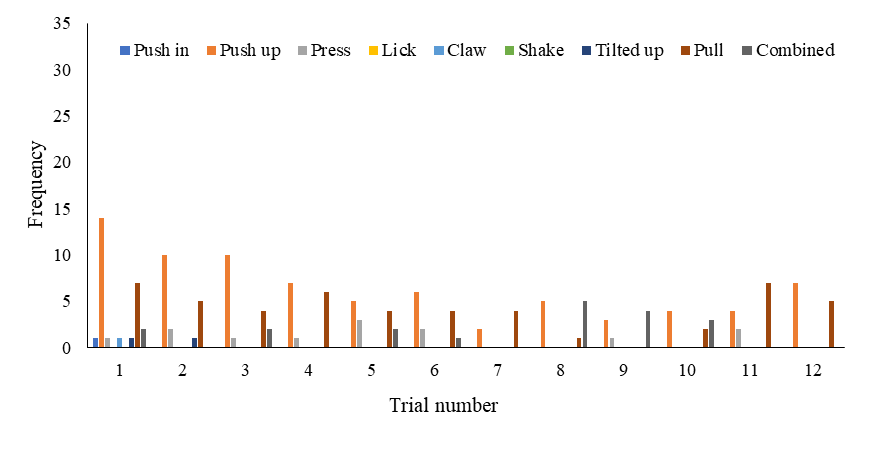


Simon


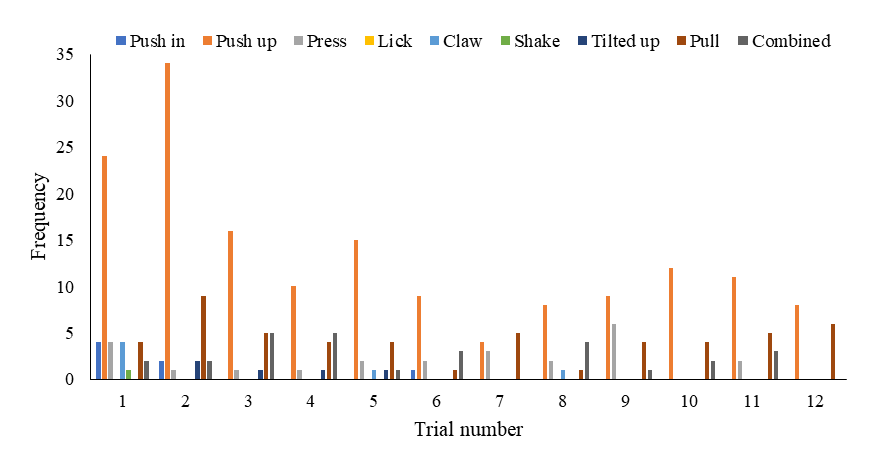
Arnold

Sarah


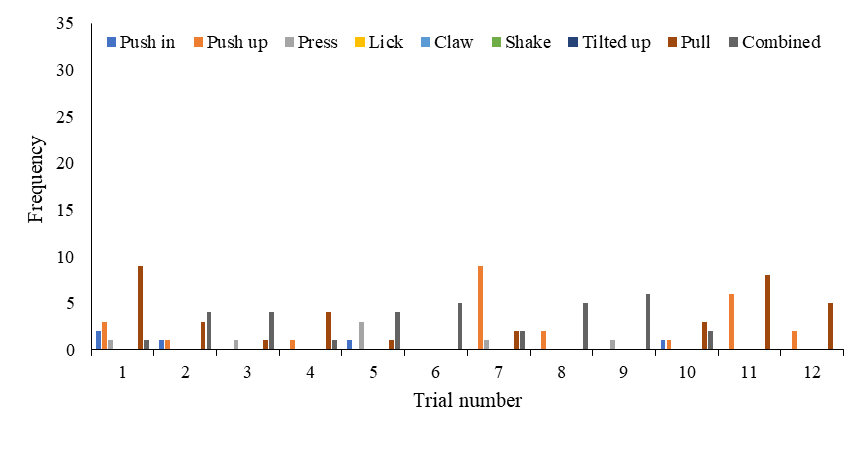


**Fig. S5.** Median, max and minimum of mean number of behaviours to each success in the original problem (grey bar) and in the modified problem (white bar).

*Analysis on the relationship between previous success and solving duration*

Spearman rank correlation (r_s_) showed the relationship between the number of successful pulling solution previously used and the mean solving duration to obtain a success in the first trial for the modified problem was not significant (p = 0.28). Despite this, there was a negative trend between the two variables of interest (r_s_ = -0.6); squirrels that had previously used the alternative successful pulling solution more showed lower mean solving duration to obtain a success in the first trial in the modified problem.

**Fig. S6.** Relationship between the number of successful pulling experience previously used and solving duration on the first trial of the modified problem.
